# Association of GLP-1 receptor agonists with risk of intestinal obstruction in patients with type 2 diabetes mellitus: a retrospective cohort study

**DOI:** 10.1007/s00592-025-02525-z

**Published:** 2025-06-05

**Authors:** Zhenxiang Gao, Tomasz Tabernacki, Ian Dorney, Pingjian Ding, David C. Kaelber, Rong Xu

**Affiliations:** 1 Center for Artificial Intelligence in Drug Discovery, School of Medicine, Case Western Reserve University, Cleveland, OH USA; 2 School of Medicine, Case Western Reserve University, Cleveland, OH USA; 3 Center for Clinical Informatics Research and Education, The Metro Health System, Cleveland, OH USA

**Keywords:** GLP-1 receptor agonists, Intestinal obstruction, Type 2 diabetes mellitus

## Abstract

**Background:**

Glucagon-like peptide-1 receptor agonists (GLP-1RAs) are approved for treating type 2 diabetes and weight loss. There have been concerns that GLP-1RAs may increase the risk of intestinal obstruction.

**Objective:**

Investigate the association between GLP-1RAs use and intestinal obstruction in a large cohort of patients with type 2 diabetes mellitus (T2DM) in the U.S.

**Design:**

A retrospective cohort study was conducted using longitudinal electronic health records sourced from TriNetX, a large-scale, population-based health database.

**Participants:**

The study included over 1.2 million T2DM patients who were prescribed anti-diabetic medications, including 181,795 prescribed GLP-1RAs between April 2013 and April 2019.

**Main measures:**

The incidence of intestinal obstruction was compared between GLP-1RAs and each of six other classes of non-GLP-1RA anti-diabetic medications. Hazard ratios (HRs) at 1, 3, and 5-year follow-up periods were calculated using Cox proportional hazards analysis. Separate analyses were performed in T2DM patients with and without obesity.

**Key results:**

The risk of intestinal obstruction did not differ between GLP-1RAs and other anti-diabetic medications except for a reduced risk compared with insulins. The results were consistent for 1, 3, and 5-year follow-up periods and in patients with and without obesity.

**Conclusions:**

Our findings do not support an increased risk of intestinal obstruction in T2DM patients prescribed GLP-1 RAs compared to other anti-diabetic medications.

**Supplementary Information:**

The online version contains supplementary material available at 10.1007/s00592-025-02525-z.

## Introduction

Glucagon-like peptide-1 receptor agonists (GLP-1RAs) are medications for managing type 2 diabetes (T2DM) and weight loss [1]. These agents mimic GLP-1, enhancing glucose-dependent insulin secretion, suppressing glucagon release, and slowing gastric emptying to improve glycemic control, as indicated by reduced HbA1c level, a crucial measure in diabetes care [2, 3]. Recently, there have been concerns of an increased risk of intestinal obstruction associated with GLP-1RAs [4–13].

Clinical trials conducted so far have not shown such changes in the human gut [14]. Observational studies have produced mixed results regarding the impact of GLP-1RAs on intestinal obstruction in patients with T2DM. For instance, a study utilizing data from the United Kingdom Clinical Practice Research Datalink (UK CPRD) suggested an increased risk of intestinal obstruction in T2DM patients prescribed GLP-1RAs compared to those prescribed sodium-glucose cotransporter-2 (SGLT2) inhibitors [15]. However, another analysis based on data from three European countries did not confirm this signal, suggesting that the increased risk associated with GLP-1RAs may not be significant compared to SGLT-2 inhibitors among T2DM patients [16]. The study using UK CPRD data, which included a small population and thus had a limited outcome sample size, may impact the statistical power to evaluate the risk. Additionally, both studies compared GLP-1RAs with SGLT-2 inhibitors, but the risk of intestinal obstruction associated with GLP-1RAs compared to other commonly used anti-diabetic medications, including insulin and metformin, remains unknown.

In this study, we used a large electronic health record (EHR) database encompassing over 1.2 million T2DM patients in the U.S. to conduct a multicenter retrospective cohort study to assess the association of GLP-1RAs with the risk of intestinal obstruction compared with non-GLP-1RA anti-diabetic medications including SGLT2 inhibitors, metformin, sulfonylureas (SU), thiazolidinediones (TZDs), dipeptidyl peptidase-4 (DPP-4) inhibitors, and insulins, across follow-up periods of 1, 3, and 5 years. We further conducted separate analyses in patients with and without obesity.

## Methods

### Data source

We used the electronic health records (EHRs) available on the TriNetX platform [17]. TriNetX provides real-time and de-identified EHR data from over 100 million patients across participating 77 healthcare organizations within the United States. The platform provides aggregated data from inpatient and outpatient clinical encounters including demographics, diagnoses, procedures, medications, laboratory tests, and genomic information. The built-in analytical functions (for example, incidence, prevalence, outcomes analysis, survival analysis, propensity score matching) allow for patient-level analyses, while only reporting population-level data to the researcher. TriNetX offers anonymized patient data, exempt from review as not Human Subject Research, therefore Institutional Review Board review was not required. The TriNetX Analytics network has been shown to be useful for large-scale cohort studies in various populations [18–24].

### Source population and study cohorts

The study population included patients with medical visits for T2DM within the TriNetX network healthcare organizations who were subsequently prescribed anti-diabetic medications between April 2013 and April 2019. GLP-1RAs considered in our study include lixisenatide, albiglutide, dulaglutide, semaglutide, liraglutide, and exenatide. The following major classes of non-GLP-1RA anti-diabetic drugs were used as comparison drugs in our study: SGLT2 inhibitors, metformin, sulfonylureas, thiazolidinediones, DPP-4 inhibitors, and insulins.

**Fig. 1:**
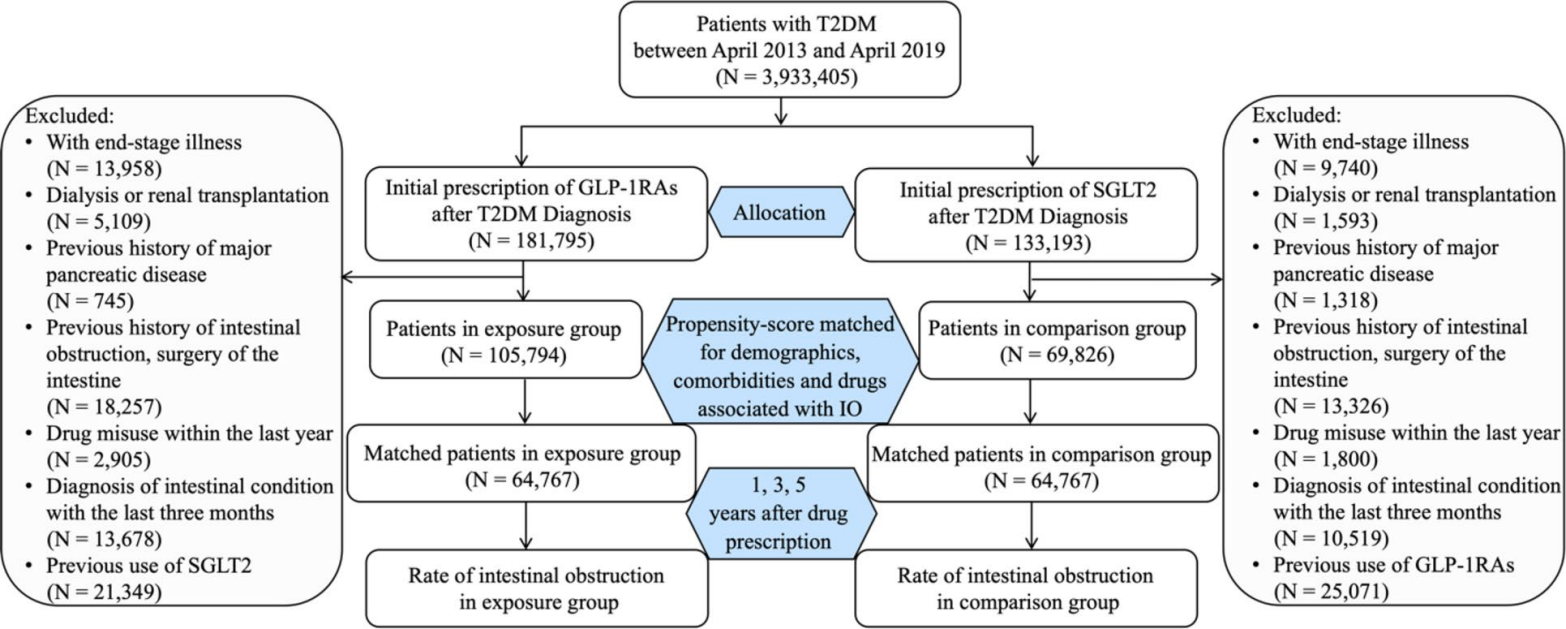
Flow diagram of cohort design (GLP-1RAs cohort vs. SGLT2 cohort)

For comparing GLP-1RAs with SGLT2 inhibitors (see Fig. 1), the study population was divided as follows: the GLP-1RA cohort consisted of patients prescribed initial GLP-1RAs between April 2013 and April 2019 who were never prescribed SGLT2 inhibitors before the initial GLP-1RAs prescription, and the SGLT2 cohort consisted of patients prescribed initial SGLT2 inhibitors between April 2013 and April 2019 who were never prescribed GLP-1RAs before the initial SGLT2 prescription. Exclusion criteria included end-stage illness, dialysis, renal transplantation, severe pancreatic disorders, bowel obstruction or major surgery of the intestines before cohort entry, the diagnosis of drug misuse within one year of cohort entry, and the diagnosis of diseases (e.g., inflammatory bowel disease, neoplasms, or hernias) that could cause constipation or intestinal obstruction within 90 days of cohort entry. Details of exclusion criteria were provided in Supplementary Table S1. Similar designs were applied to compare GLP-1RAs with metformin, DPP-4 inhibitors, sulfonylureas, thiazolidinediones, and insulins. Flow diagrams of these cohort designs were provided in in Supplementary Figure S1-S5.

Overweight and obesity significantly increase the risk of various gastrointestinal disorders [25], and they often co-occur with T2DM in patients. We performed sensitivity analyses to evaluate the impact of GLP-1RAs on the risk of intestinal obstruction compared to other anti-diabetic medications in patients with T2DM, stratified by obesity status. For the analysis involving patients with both T2DM and obesity, we included those who were prescribed GLP-1RAs or control medications within one year following their diagnosis of both T2DM and obesity, between April 2013 and April 2019. In assessing the risk in T2DM patients without obesity, we included individuals diagnosed with type 2 diabetes (T2DM) who had never been diagnosed with obesity.

### Outcomes

The outcome of interest, intestinal obstruction, was identified using ICD-10 diagnosis codes, including obstruction of duodenum (ICD K31.5), paralytic ileus (ICD K56.0), ileus (ICD K56.7), and other specified types of intestinal obstruction (ICD K56.4, K56.6) [15, 16]. Details of the ICD codes for intestinal obstruction are listed in Supplementary Table S2. Sensitivity analyses were further conducted for each subtype of intestinal obstruction.

### Covariates

Each patient in the exposure group was matched to a patient in the control group using propensity-score matching [26]. The list of covariates matched is included in Supplementary Table S3. These covariates included demographics (age, gender [male, female, unknown], race [Black or African American, White, Asian], ethnicity [Hispanic or Latino, Not Hispanic or Latino, unknown], and marital status), socioeconomic determinants of health (SDOHs) including education, employment, environmental exposure, and housing, problems related to lifestyle, cancer, diabetes severity indicators (e.g., obesity, complications like nephropathy, liver, neuropathy, heart disease), some conditions known to be associated with constipation or intestinal obstruction (e.g., diseases of intestines, diseases of appendix), and prior prescription of medicines associated with diabetes, decreased intestinal motility, or constipation [15, 16, 27–30].

### Statistical analysis

The comparison of risk among the cohorts was performed using Cox proportional hazards regression analysis, focusing on the incidence at 1, 3, and 5 years following the prescription date of the drugs. Patients were censored at outcomes, death, loss of follow-up, or end of follow-up. Hazard ratios (HR) along with 95% confidence intervals (CI) were used to compare the time to the event rates of intestinal obstruction. All statistical analyses were conducted on the TriNetX Advanced Analytics Platform.

## Results

### Risks of intestinal obstruction in patients with T2DM: GLP-1 RAs vs. non-GLP-1RA Anti-diabetic medications

**Fig. 2:**
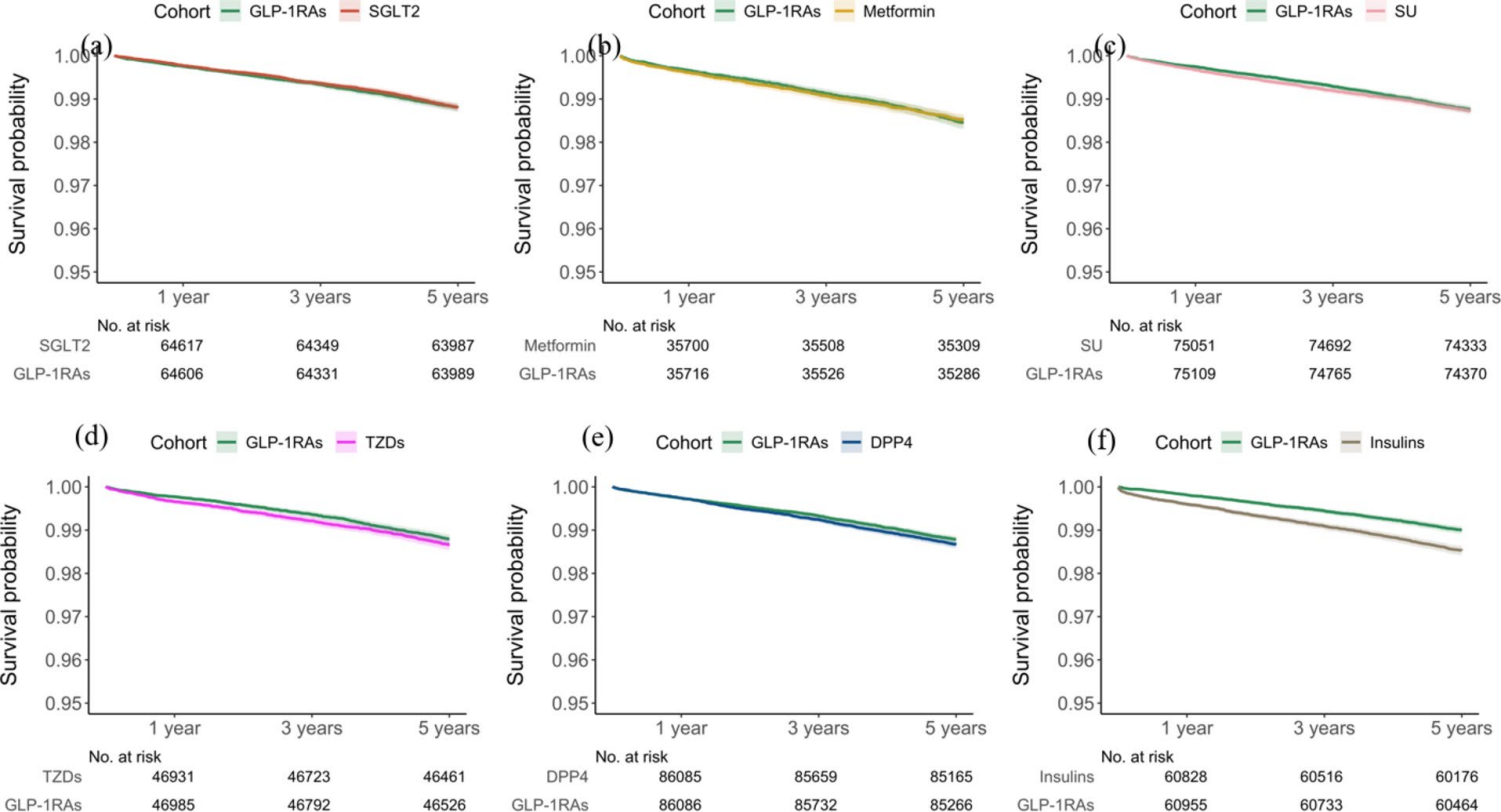
Kaplan-Meier plots illustrating intestinal obstruction-free survival in T2DM patients prescribed with GLP-1RAs compared with those prescribed with (**a**) SGLT2 inhibitors, (**b**) metformin, (**c**) sulfonylureas, (**d**) thiazolidinediones, (**e**) DPP-4 inhibitors, or (**f**) insulins at 1-year, 3-year, and 5-year follow-up periods

**Fig. 3:**
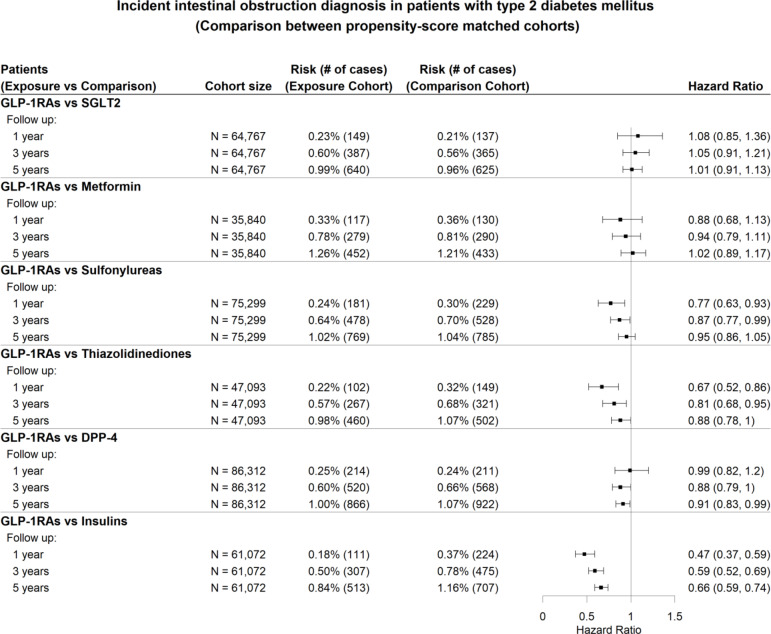
Hazard ratios for the diagnosis of intestinal obstruction in T2DM patients prescribed with GLP-1RAs compared with propensity score-matched patients prescribed alternative diabetes medications

The study first separately compared GLP-1RAs with each of the six classes of non-GLP-1RA anti-diabetic medications (SGLT2 inhibitors, metformin, sulfonylureas, thiazolidinediones, DPP-4 inhibitors, insulins). In a comparison between GLP-1RAs and SGLT2 inhibitors, 105,794 patients were included in the GLP-1RA cohort, and 69,826 patients were in the SGLT2 inhibitor cohort. Patient characteristics were detailed in Table 1. The GLP-1RA cohort compared with the SGLT2 cohort included more women, more Black people, had higher SDOHs and prevalence of thyroid disorders, kidney disease, and prior prescriptions of opioids, diuretics, antihistamines, and antidepressants. After propensity score matching, the two cohorts were balanced. The Kaplan-Meier curve for the study cohort with the outcome of intestinal obstruction was shown in Fig. 2. Our analysis found no significant increase in the incidence of intestinal obstruction in the GLP-1RA group compared to the SGLT2 inhibitors and metformin groups (Fig. 3). When comparing GLP-1RAs with sulfonylureas or thiazolidinediones, lower risks of intestinal obstruction were observed at one and three years of follow-up, but no associations were observed after five years of follow up. GLP-1RAs was significantly associated with decreased risk of intestinal obstruction compared to DPP-4 inhibitors after five years of follow-up, but the associations were not statistically significant at one and three years. Patients prescribed GLP-1RAs displayed a significantly reduced hazard ratio for the diagnosis of intestinal obstruction compared with patients prescribed insulin at 1, 3, and 5 years. Subgroup analyses for elderly patients, along with sensitivity analyses conducted for covariates and each subtype of intestinal obstruction, confirmed results consistent with the primary findings. Detailed results are provided in Figures S6–S8 of the supplementary file.

**Table 1:** Characteristics of patients with type-2 diabetes in the GLP-1RAs (exposure) and SGLT2 inhibitors (control) cohorts before and after propensity-score matching

Characteristics	Before matching			After matching		
	Exposure cohort	Comparison cohort	SMD	Exposure cohort	Comparison cohort	SMD
Total No.	105,794	69,826		64,767	64,767	
Age	56.1(12.2)	57.4(11.5)	0.11*	57(11.9)	57(11.5)	0.0008
Sex, %						
Female	53.7	42.3	0.23*	44.6	44.6	0.0002
Male	42.2	52.6	0.21*	50.3	50.3	0.0002
Unknown Gender	4.1	5.1	0.05	5.1	5.1	< 0.0001
Ethnicity, %						
Hispanic/Latinx	9.8	11.2	0.04	10.8	10.8	0.0004
Not Hispanic/Latinx	69.2	66.1	0.06	66.6	66.5	0.001
Unknown Ethnicity	21.0	22.7	0.04	22.6	22.7	0.002
Race, %						
African American/Black	15.8	11.7	0.11*	12.4	12.4	0.0006
White	65.3	66.3	0.02	66.7	66.8	0.001
Asian	2.2	4.2	0.11*	3.2	3.1	0.007
Adverse socioeconomic determinants of health, %	1.8	1.3	0.04	1.4	1.3	0.002
Problems related to lifestyle, %	3.4	2.9	0.02	2.9	2.9	0.001
Pre-existing medical conditions, %						
Ischemic heart diseases	16.1	17.4	0.03	16.6	16.4	0.006
Other forms of heart disease	19.2	17.7	0.03	17.3	17.4	0.001
Cerebrovascular diseases	6.1	5.9	0.006	5.8	5.7	0.004
Disorders of thyroid gland	18.4	15.1	0.08	15.7	15.5	0.003
Diseases of liver	6.2	5.2	0.03	5.3	5.3	0.001
Diseases of arteries, arterioles and capillaries	8.1	7.3	0.03	7.3	7.2	0.002
Diseases of veins, lymphatic vessels and lymph nodes, not elsewhere classified	6.9	5.2	0.07	5.5	5.4	0.004
Disorders of gallbladder, biliary tract and pancreas	3.6	3.2	0.02	3.2	3.2	0.0008
Diseases of appendix	0.17	0.16	0.001	0.15	0.15	0.0007
Acute kidney failure and chronic kidney disease	11.4	6.2	0.18*	6.8	6.6	0.007
Renal tubulo-interstitial diseases	2.8	2.2	0.04	2.2	2.2	0.004
Glomerular diseases	2.4	1.3	0.07	1.4	1.4	0.001
Urolithiasis	4.9	4.2	0.03	4.1	4.2	0.004
Hyperlipidemia	48.1	45.8	0.04	45.1	45.2	0.001
Gastroparesis	0.8	0.7	0.01	0.7	0.7	0.0009
Anorexia nervosa	0.03	0.01	0.01	0.02	0.02	0
Disorders of fluid, electrolyte and acid-base balance	8.3	6.3	0.07	6.4	6.4	0
Malignant neoplasms of digestive organs	0.3	0.3	0.004	0.3	0.3	0.003
Intestinal infectious diseases	1.5	1.1	0.03	1.1	1.1	0.003
Noninfective enteritis and colitis	6.0	4.6	0.06	4.7	4.7	0.0008
Hernia	4.1	3.5	0.03	3.5	3.6	0.0008
Diseases of peritoneum and retroperitoneum	0.9	0.6	0.03	0.6	0.6	0.007
Overweight and obesity	41.2	28.6	0.26*	30.3	30.4	0.002
Pre-existing procedures, %						
Surgical Procedures on the Colon and Rectum	0.85	0.08	0.11*	0.08	0.09	0.002
Pre-existing anti-diabetic medicine, %						
Insulins	43.6	30.3	0.27*	32.2	32.1	0.002
Biguanides	58.3	56.6	0.03	55.2	55.5	0.005
Sulfonylureas	32.2	31.3	0.01	30.9	30.7	0.004
Alpha glucosidase inhibitors	0.54	0.43	0.01	0.46	0.4	0.003
Thiazolidinediones	9.7	7.9	0.06	8.1	7.9	0.004
Dipeptidyl peptidase 4 (DPP-4) inhibitors	19.5	24.4	0.11*	22.2	22.1	0.002
Other blood glucose lowering drugs, excl. insulins	2.2	1.6	0.03	1.6	1.7	0.004
Pre-existing other medicine, %						
ACE inhibitors	38.9	36.1	0.05	35.7	35.7	0.0007
Anti-inflammatory and antirheumatic products	35.6	29.9	0.12	30.1	30.3	0.002
Opioids	34.7	29.8	0.11*	30.2	29.9	0.004
Glucocorticoids	34.4	29.7	0.11*	29.9	29.8	0.002
Diuretics	35.8	29.3	0.14*	30.1	29.9	0.002
Beta blocking agents	31.1	28.5	0.05	28.3	28.1	0.002
Platelet aggregation inhibitors	29.6	27.4	0.04	26.9	26.9	0.0003
Antihistamines	28.4	22.7	0.13*	23.2	23.1	0.002
Antidepressants	28.7	21.1	0.17*	22.1	22.0	0.002
Serotonin (5HT3) antagonists	22.9	19.7	0.07	19.9	19.8	0.002
Drugs for constipation	23.4	18.6	0.11*	19.1	19.0	0.001
Calcium channel blockers	21.1	18.4	0.06	18.3	18.4	0.002
Selective beta-2-adrenoreceptor agonists	21.9	16.9	0.12*	17.5	17.5	0.0006
Anticoagulants	18.4	16.1	0.05	16.1	16.1	0.001
Anxiolytics	18.1	14.5	0.09	14.9	14.8	0.005
Anticholinergics	7.6	5.7	0.07	5.8	5.9	0.0003
Antipsychotics	5.5	3.8	0.07	4.1	4.0	0.002

*SMD* standardized mean difference. *SMD > 0.1, a threshold indicating imbalance between cohorts

### Risks of intestinal obstruction in T2DM patients with and without obesity

**Fig. 4:**
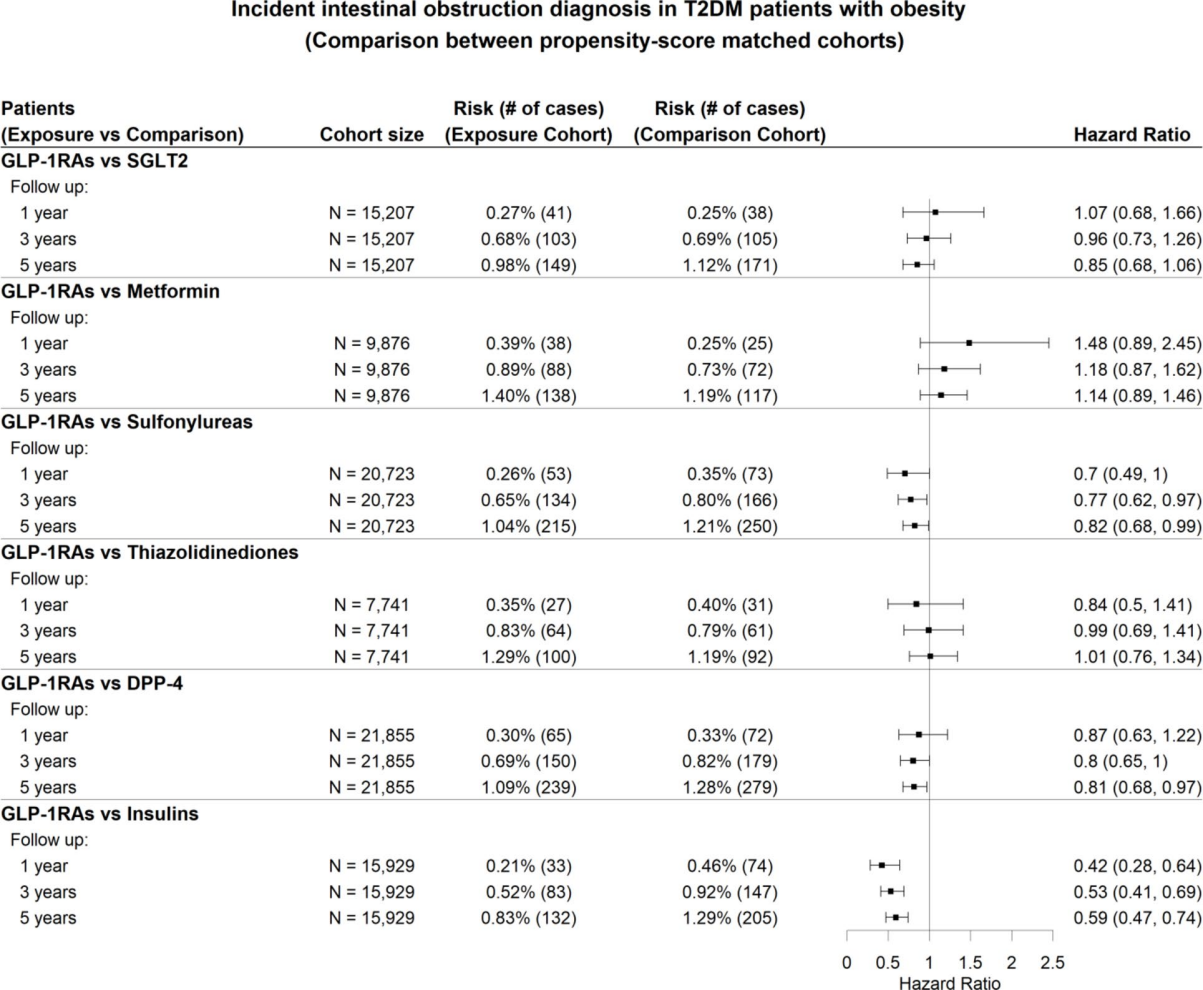
Hazard ratios for the diagnosis of intestinal obstruction in patients with T2DM and obesity who were prescribed GLP-1RAs, compared to propensity score-matched patients who were prescribed six other classes of anti-diabetic medications

**Fig. 5:**
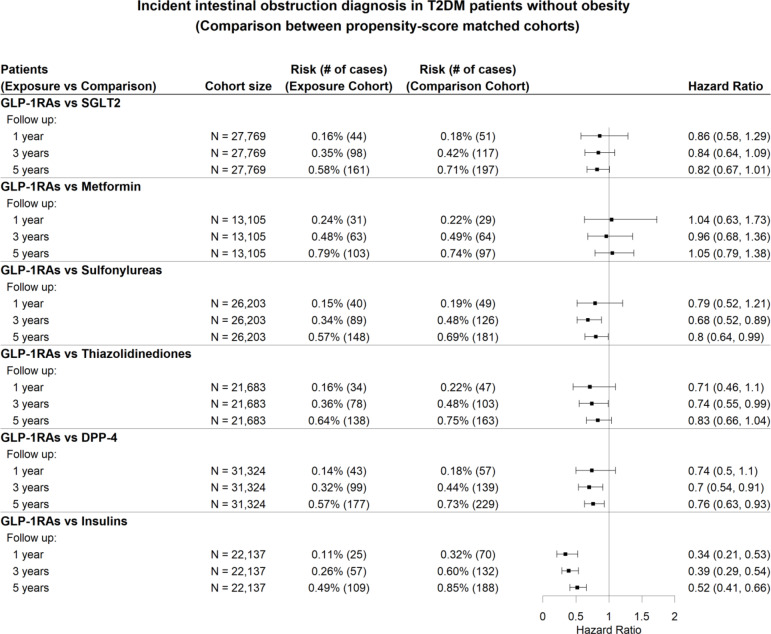
Hazard ratios for the diagnosis of intestinal obstruction in T2DM patients without obesity prescribed GLP-1RAs, in comparison to propensity score-matched patients who were prescribed six other classes of anti-diabetic medications

We performed stratified analyses of the incidence of intestinal obstruction among T2DM patients with and without obesity. The detailed patient characteristics for the GLP-1RAs and SGLT2 inhibitor cohorts are provided in Supplementary Table S4-5. In patients with obesity, GLP-1RAs were not associated with a significantly increased risk of intestinal obstruction in patients with T2DM and obesity when compared to other anti-diabetic medications, except for insulin (Fig. 4). Similar findings were observed in patients without obesity (Fig. 5). The sensitivity analyses for each subtype of intestinal obstruction yielded results consistent with the findings above. For details, refer to Figure S9-S10 in the supplementary file.

## Discussion

Our study found that GLP-1RAs were not associated with an increased risk of intestinal obstruction incidence in patients with T2DM, compared with six other anti-diabetic medications. These findings were consistent in patients with and without obesity.

GLP-1RAs bind to the Glucagon-like peptide-1 (GLP-1) receptor to stimulate the pancreas to release insulin in response to in response to hyperglycemia, effectively lowering glucose levels [31]. GLP-1 is essential for maintaining normal glucose homeostasis, and its increased action reduces gastrointestinal motility [10, 32]. Due to this effect, there has been a concern regarding the risk of intestinal obstruction with the use of GLP-1RAs. Several retrospective cohort studies have explored the association between the use of GLP-1RAs and the risk of intestinal obstruction compared to SGLT2 inhibitors in T2DM patients, with mixed outcomes. Faillie et al., utilizing the UK CPRD database, found an increased risk of intestinal obstruction with GLP-1RAs compared to SGLT-2 inhibitors [15]. The small number of outcome events in this study, which recorded only 70 events among 25,617 users of GLP-1RAs and had only 6,622 patients on GLP-1RAs remaining two years after cohort entry, has limited statistical power. In contrast, a recent large registry cohort study by Ueda et al., which utilized data from nationwide registers in Sweden, Denmark, and Norway, found no difference in the risk of intestinal obstruction when comparing GLP-1RAs to SGLT2 inhibitors among over 300,000 patients [16]. Our retrospective cohort study showed results consistent with Ueda’s work, indicating that GLP-1RAs were not associated with a significant risk of intestinal obstruction compared to SGLT2 inhibitors.

Furthermore, our study expanded the comparison to five other classes of anti-diabetic medications. Metformin, a first-line T2DM treatment, affects the gastrointestinal tract by altering glucose uptake, GLP-1 secretion, gut microbiota, and immune response [33, 34], but it rarely causes bowel obstruction. Sulfonylureas [35], which lower blood glucose by stimulating insulin secretion, and thiazolidinediones [36], which act as insulin sensitizers by binding to PPAR-γ receptors, are similarly not commonly associated with intestinal obstruction. DPP-4 inhibitors [37], which elevate incretin hormone levels to maintain glucose homeostasis, have yielded mixed findings regarding their association with intestinal obstruction: a smaller study of 40,615 patients indicated a potential risk [15], while a larger cohort study of 190,321 patients across three countries found no increased risk [16]. Clinical trials indicated that the levels of active GLP-1 achieved through DPP-4 inhibition might not be sufficient or sustained enough to significantly impact gastrointestinal motility [38]. In comparing GLP-1RAs with these medications, no significant difference in intestinal obstruction risk was observed. Insulin [39], an essential hormone that helps the body convert food into energy and regulate blood sugar levels, rarely causes gastrointestinal distress, and any such effects generally improve with dose adjustment [40]. We found that GLP-1RAs were associated with a reduced risk of intestinal obstruction compared to insulin. Diabetes-related factors, including neuropathy affecting nerves that control the gastrointestinal tract [41, 42] and changes in gut motility [43], can lead to slowed digestion, constipation, and potential bowel obstruction. Patients prescribed insulin may have a longer history of diabetes and more severe complications [44], which could increase their risk of gastrointestinal issues. Since insulin therapy often indicates more advanced disease progression, greater severity, or additional comorbidities, which may lead to a higher risk of intestinal obstruction, and there was no statistically significant difference in intestinal obstruction risk between GLP-1RAs and other anti-diabetic medications (e.g., SGLT2 inhibitors, metformin, sulfonylureas, thiazolidinediones, DPP-4 inhibitors), the observed reduction relative to insulin does not necessarily suggest a protective effect of GLP-1RAs against intestinal obstruction. However, these findings strongly support that GLP-1RAs do not increase the risk of intestinal obstruction compared to non-GLP-1RA anti-diabetic medications.

Overweight and obesity is a significant risk factor for T2DM and significantly impacts gastrointestinal health, leading to various adverse events [25]. GLP-1RAs aid in weight loss by reducing the appetite and feelings of hunger, slowing the stomach empties, and increasing feelings full for longer. In a previous cohort study comparing GLP-1RAs with anti-obesity medications bupropion-naltrexone in patients with obesity who had no T2DM [45] showed an increased risk of bowel obstruction. Our study comparing GLP-1RAs with other anti-diabetes medications in T2DM patients with and without obesity, showed no increased risk of bowel obstruction. These results suggest that the any potential associations between GLP-1RAs and intestinal obstruction in patients with T2DM may not entirely mediate through weight loss.

Our study has also several limitations. First, this was a retrospective observational study of patient electronic health records, which have inherent limitations in misdiagnosis, overdiagnosis, underdiagnosis, unmeasured or uncontrolled confounders, and biases, so no causal inferences can be drawn. Second, we evaluated GLP-1RAs as a class, comparing them with other anti-diabetic medication classes, without assessing individual GLP-1RAs, primarily due to limited sample sizes. Future research is warranted to examine potential differential effects among specific GLP-1RA medications. Second, the study focused exclusively on T2DM patients during the period from April 2013 to April 2019, with up to five years of follow-up. During this time, all GLP-1RAs were approved for T2DM, and the analysis was therefore limited to comparing GLP-1RAs with other anti-diabetic medications in this population. Future work will be necessary to perform similar studies in patients with obesity by comparing new generation anti-obesity GLP-1RAs with other anti-obesity medications in patients with obesity. Third, we could not establish the dose-response relationship of GLP-1RAs on outcomes due to sample size limitations. Additionally, the information available in the EHR database provides limited details on drug usage and patient adherence. For example, patients may obtain prescriptions from providers outside of the TriNetX network, and these records may not be captured in our analysis. Moreover, patients may be prescribed a drug but may not be taking it as prescribed. Consequently, we were unable to thoroughly evaluate how factors like drug usage duration, compliance might influence the development of the condition under investigation. Furthermore, our study utilized an intention-to-treat approach [46, 47], comparing the effects of being assigned to the treatment strategies at baseline, regardless of whether participants followed these strategies throughout the follow-up period. Fourth, the potential underreporting or inconsistent registration of obesity in EHRs may affect an incomplete assessment of obesity as a contributing factor in our study. Fifth, the data may not encompass all potential confounding factors that could influence the outcome of interest. To address these concerns, we employed propensity-score matching to balance known confounding variables. However, biases and residual confounding inherent to EHR-based research could not be eliminated. Finally, we were unable to explicitly control for healthcare utilization and insurance type. Furthermore, we could not directly control for disease severity and management quality of medical conditions, including diabetes duration, glycemic control, body mass index, and lipid profile. These factors could have confounded our findings.

In conclusion, the findings from this study show that GLP-1RAs were not associated with an increased risk of intestinal obstruction in patients with T2DM. Findings from this study may provide real-world evidence contributing to our knowledge of safety profile of GLP-1RAs, especially in terms of their impact on gastrointestinal functions.

## Electronic supplementary material

Below is the link to the electronic supplementary material.

Supplementary Material 1

Supplementary Material 2

## Data Availability

The EHR database used in this study were obtained from TriNetX (https://trinetx.com/).

## Abbreviations

GLP-1RAs: Glucagon-like peptide-1 receptor agonists
T2DM: Type 2 diabetes mellitus
EHRs: Electronic health records
SGLT2: Sodium-glucose cotransporter-2
DPP-4: Dipeptidyl peptidase-4
HR: Hazard ratios
CI: Confidence intervals
