# Supplementary material for: Association of GLP-1 receptor agonists with risk of intestinal obstruction in patients with type 2 diabetes mellitus: a retrospective cohort study
Source: Acta Diabetol. 2025 Jun 5;62(11):1941–51. doi: 10.1007/s00592-025-02525-z (PMC12640328; doi:10.1007/s00592-025-02525-z)

Supporting Information

**Table S1**. Names and codes of exclusion criteria

| **Criteria** | **Name** | **code** |
| --- | --- | --- |
| End-stage illness | Malnutrition | E40-E43 |
|  | dementia | F01-F02, G30 |
|  | Coma | R40.2 |
|  | Cachexia | R64 |
| Dialysis or renal transplantation | Dialysis or renal transplantation | Z49,Z94.0,Z99.2,CPT 1008098 |
| Major pancreatic disease | Major pancreatic disease | C25, K86.0, K86.1, 1007908 |
| Intestinal obstruction, surgery of the intestine | Intestinal obstruction | K56, K31.5, K59.2, K59.3, |
|  | Intestine surgery | CPT 1007422, 1007570, 1007578, 1007591 |
| Drug misuse | Drug misuse | R78.1-R78.5, F11-F19, T40 |
| Condition associated with Intestinal obstruction | Intestinal infectious diseases | A00-A09 |
|  | Malignant neoplasms of digestive organs | C15-C26 |
|  | Malignant neoplasm of retroperitoneum and peritoneum | C48 |
|  | Malignant neoplasm of connective and soft tissue of abdomen | C49.4 |
|  | Malignant neoplasm of connective and soft tissue of pelvis | C49.5 |
|  | Diseases of appendix | K35-K38 |
|  | Hernia | K40-K46 |
|  | Noninfective enteritis and colitis | K50-K52 |
|  | Other diseases of intestines | K55-K64 |
|  | Diseases of peritoneum and retroperitoneum | K65-K68 |
|  | Diseases of liver | K70-K77 |
|  | Disorders of gallbladder, biliary tract and pancreas | K80-K87 |
|  | Other diseases of the digestive system | K90-K95 |

**Table S2.** ICD codes of Intestinal obstruction

| **ICD code** | **Item name** |
| --- | --- |
| K31.5 | Obstruction of duodenum |
| K56.0 | Paralytic ileus |
| K56.4 | Other impaction of intestine |
| K56.6 | Other and unspecified intestinal obstruction |
| K56.7 | Ileus, unspecified |

**Table S3**. Covariates and their standardized names and codes

| **Covariate** | **Description** | **abbreviation or code** |
| --- | --- | --- |
| Age | Age at index | AI |
| Sex | Female | F |
|  | Male | M |
|  | Unknown Gender | UN |
| Ethnicity | Hispanic/Latinx | 2135-2 |
|  | Not Hispanic/Latinx | 2186-5 |
|  | Unknown Ethnicity | UN |
| Race | African American/Black | 2054-5 |
|  | White | 2106-3 |
|  | Asian | 2028-9 |
| Socioeconomic status | Persons with potential health hazards related to socioeconomic and psychosocial circumstances | Z55-Z65 |
| Problems related to lifestyle | Problems related to lifestyle | Z72 |
| Pre-existing medical conditions | Ischemic heart diseases | I20-I25 |
|  | Other forms of heart disease | I30-I5A |
|  | Cerebrovascular diseases | I60-I69 |
|  | Disorders of thyroid gland | E00-E07 |
|  | Diseases of liver | K70-K77 |
|  | Diseases of arteries, arterioles and capillaries | I70-I79 |
|  | Diseases of veins, lymphatic vessels and lymph nodes, not elsewhere classified | I80-I89 |
|  | Disorders of gallbladder, biliary tract and pancreas | K80-K87 |
|  | Diseases of appendix | K35-K38 |
|  | Acute kidney failure and chronic kidney disease | N17-N19 |
|  | Renal tubulo-interstitial diseases | N10-N16 |
|  | Glomerular diseases | N00-N08 |
|  | Urolithiasis | N20-N23 |
|  | Hyperlipidemia | E78.5 |
|  | Gastroparesis | K31.84 |
|  | Anorexia nervosa | F50.0 |
|  | Other disorders of fluid, electrolyte and acid-base balance | E87 |
|  | Malignant neoplasms of digestive organs | C15-C26 |
|  | Intestinal infectious diseases | A00-A09 |
|  | Noninfective enteritis and colitis | K50-K52 |
|  | Hernia | K40-K46 |
|  | Diseases of peritoneum and retroperitoneum | K65-K68 |
|  | Overweight and obesity | E66 |
| Pre-existing procedures | Surgical Procedures on the Colon and Rectum | 1007591 |
| Pre-existing anti-diabetes medicine | Insulins | A10A |
|  | Biguanides | A10BA |
|  | Sulfonylureas | A10BB |
|  | Alpha glucosidase inhibitors | A10BF |
|  | Thiazolidinediones | A10BG |
|  | Dipeptidyl peptidase 4 (DPP-4) inhibitors | A10BH |
|  | Other blood glucose lowering drugs, excl. insulins | A10BX |
| Pre-existing other medicine | ACE inhibitors | CV800 |
|  | Anti-inflammatory and antirheumatic products | M01A |
|  | Opioids | N02A |
|  | Glucocorticoids | H02AB |
|  | Diuretics | CV700 |
|  | Beta blocking agents | C07 |
|  | Platelet aggregation inhibitors | BL117 |
|  | Antihistamines | AH000 |
|  | Antidepressants | CN600 |
|  | Serotonin (5HT3) antagonists | A04AA |
|  | Drugs for constipation | A06 |
|  | Calcium channel blockers | CV200 |
|  | Selective beta-2-adrenoreceptor agonists | R03AC |
|  | Anticoagulants | BL110 |
|  | Anxiolytics | N05B |
|  | Anticholinergics | R03BB |
|  | Antipsychotics | CN700 |

**Table S4**. Characteristics of T2DM patients with obesity in the GLP-1RAs (exposure) and SGLT2 inhibitors (control) cohorts before and after propensity-score matching.

| **Characteristics** | **Before Matching** | | | **After Matching** | | |
| --- | --- | --- | --- | --- | --- | --- |
|  | **Exposure Cohort** | **Comparison Cohort** | **SMD** | **Exposure Cohort** | **Comparison Cohort** | **SMD** |
| Total No. | 31,462 | 15,345 |  | 15,207 | 15,207 |  |
| Age | 53.3(12.4) | 54.6(11.7) | 0.11* | 54.5(12) | 54.5(11.7) | 0.001 |
| ***Sex, %*** | | | | | | |
| Female | 56.4 | 45.6 | 0.21* | 45.8 | 45.9 | 0.003 |
| Male | 39.1 | 48.9 | 0.19* | 48.7 | 48.5 | 0.003 |
| Unknown Gender | 4.5 | 5.5 | 0.04 | 5.5 | 5.6 | 0.001 |
| ***Ethnicity, %*** | | | | | | |
| Hispanic/Latinx | 10.9 | 12.3 | 0.04 | 12.3 | 12.2 | 0.001 |
| Not Hispanic/Latinx | 67.7 | 66.6 | 0.02 | 66.2 | 66.6 | 0.009 |
| Unknown Ethnicity | 21.4 | 21.1 | 0.006 | 21.5 | 21.3 | 0.009 |
| ***Race, %*** | | | | | | |
| African American/Black | 18.2 | 13.8 | 0.12* | 13.7 | 13.9 | 0.003 |
| White | 62.1 | 66.4 | 0.09 | 66.4 | 66.2 | 0.004 |
| Asian | 1.7 | 2.2 | 0.03 | 2.2 | 2.1 | 0.001 |
| ***Adverse socioeconomic determinants of health, %*** | 2.6 | 2.1 | 0.02 | 2.2 | 2.1 | 0.003 |
| ***Problems related to lifestyle, %*** | 4.7 | 4.3 | 0.01 | 4.3 | 4.4 | 0.002 |
| ***Pre-existing medical conditions, %*** | | | | | | |
| Ischemic heart diseases | 17.1 | 18.9 | 0.04 | 18.8 | 18.6 | 0.004 |
| Other forms of heart disease | 23.1 | 22.3 | 0.01 | 22.1 | 22.2 | 0.001 |
| Cerebrovascular diseases | 6.1 | 6.2 | 0.004 | 6.0 | 6.1 | 0.003 |
| Disorders of thyroid gland | 19.7 | 17.5 | 0.05 | 17.4 | 17.6 | 0.007 |
| Diseases of liver | 7.7 | 7.4 | 0.01 | 7.5 | 7.4 | 0.0002 |
| Diseases of arteries, arterioles and capillaries | 9.1 | 8.2 | 0.03 | 8.3 | 8.1 | 0.004 |
| Diseases of veins, lymphatic vessels and lymph nodes, not elsewhere classified | 9.4 | 8.07 | 0.04 | 8.0 | 8.1 | 0.001 |
| Disorders of gallbladder, biliary tract and pancreas | 4.2 | 4.2 | 0.004 | 4.0 | 4.1 | 0.009 |
| Diseases of appendix | 0.18 | 0.18 | 0.0003 | 0.16 | 0.18 | 0.006 |
| Acute kidney failure and chronic kidney disease | 14.0 | 8.8 | 0.16* | 8.7 | 8.8 | 0.004 |
| Renal tubulo-interstitial diseases | 3.4 | 2.7 | 0.04 | 2.9 | 2.8 | 0.001 |
| Glomerular diseases | 2.9 | 2.1 | 0.05 | 2.1 | 2.0 | 0.007 |
| Urolithiasis | 5.4 | 5.0 | 0.01 | 5.1 | 5.0 | 0.002 |
| Hyperlipidemia | 52.9 | 54.6 | 0.03 | 54.1 | 54.3 | 0.005 |
| Gastroparesis | 0.85 | 0.94 | 0.009 | 0.92 | 0.92 | 0 |
| Anorexia nervosa | 0.03 | 0 | 0.02 | 0.07 | 0 | 0.03 |
| Disorders of fluid, electrolyte and acid-base balance | 11.2 | 9.1 | 0.07 | 8.7 | 9.1 | 0.01 |
| Malignant neoplasms of digestive organs | 0.27 | 0.31 | 0.007 | 0.31 | 0.31 | 0 |
| Intestinal infectious diseases | 1.9 | 1.5 | 0.03 | 1.4 | 1.5 | 0.003 |
| Noninfective enteritis and colitis | 6.7 | 5.9 | 0.03 | 5.9 | 5.9 | 0.0008 |
| Hernia | 4.9 | 4.8 | 0.005 | 4.8 | 4.8 | 0.001 |
| Diseases of peritoneum and retroperitoneum | 1.21 | 0.83 | 0.03 | 0.83 | 0.84 | 0.001 |
| ***Pre-existing procedures, %*** | | | | | | |
| Surgical Procedures on the Colon and Rectum | 0% | 0% |  | 0% | 0% |  |
| ***Pre-existing anti-diabetic medicine, %*** | | | | | | |
| Insulins | 46.3 | 36.5 | 0.20* | 36.5 | 36.7 | 0.005 |
| Biguanides | 60.5 | 61.3 | 0.01 | 60.2 | 61.1 | 0.01 |
| Sulfonylureas | 30.4 | 31.9 | 0.03 | 31.9 | 31.7 | 0.003 |
| Alpha glucosidase inhibitors | 0.46 | 0.42 | 0.006 | 0.44 | 0.42 | 0.003 |
| Thiazolidinediones | 7.2 | 7.5 | 0.01 | 7.4 | 7.4 | 0.001 |
| Dipeptidyl peptidase 4 (DPP-4) inhibitors | 18.2 | 23.6 | 0.13* | 22.7 | 23.1 | 0.009 |
| Other blood glucose lowering drugs, excl. insulins | 1.5 | 1.5 | 0.001 | 1.5 | 1.4 | 0.002 |
| ***Pre-existing other medicine, %*** | | | | | | |
| ACE inhibitors | 40.5 | 39.9 | 0.01 | 39.3 | 39.8 | 0.009 |
| Anti-inflammatory and antirheumatic products | 50.0 | 36.7 | 0.08 | 36.4 | 36.9 | 0.006 |
| Opioids | 39.3 | 35.8 | 0.07 | 35.3 | 35.8 | 0.01 |
| Glucocorticoids | 38.9 | 35.6 | 0.06 | 34.8 | 35.6 | 0.01 |
| Diuretics | 39.9 | 36.1 | 0.07 | 36.0 | 36.2 | 0.005 |
| Beta blocking agents | 33.0 | 31.7 | 0.02 | 31.1 | 31.6 | 0.009 |
| Platelet aggregation inhibitors | 30.1 | 29.5 | 0.01 | 29.3 | 29.4 | 0.0008 |
| Antihistamines | 32.7 | 28.1 | 0.09 | 27.7 | 28.2 | 0.01 |
| Antidepressants | 31.6 | 25.2 | 0.14* | 25.2 | 25.4 | 0.004 |
| Serotonin (5HT3) antagonists | 27.1 | 24.7 | 0.05 | 24.3 | 24.7 | 0.009 |
| Drugs for constipation | 27.5 | 23.4 | 0.09 | 22.7 | 23.5 | 0.01 |
| Calcium channel blockers | 23.0 | 21.6 | 0.03 | 21.5 | 21.6 | 0.001 |
| Selective beta-2-adrenoreceptor agonists | 26.1 | 22.3 | 0.08 | 21.6 | 22.4 | 0.01 |
| Anticoagulants | 22.1 | 20.7 | 0.03 | 20.3 | 20.6 | 0.008 |
| Anxiolytics | 19.8 | 17.5 | 0.05 | 17.4 | 17.5 | 0.001 |
| Anticholinergics | 9.8 | 8.1 | 0.06 | 7.7 | 8.2 | 0.01 |
| Antipsychotics | 6.4 | 4.8 | 0.06 | 5.1 | 4.9 | 0.01 |

Note: SMD - standardized mean difference. *SMD > 0.1, a threshold indicating imbalance between cohorts.

**Table S5**. Characteristics of T2DM patients without obesity in the GLP-1RAs (exposure) and SGLT2 inhibitors (control) cohorts before and after propensity-score matching.

| **Characteristics** | **Before Matching** | | | **After Matching** | | |
| --- | --- | --- | --- | --- | --- | --- |
|  | **Exposure Cohort** | **Comparison Cohort** | **SMD** | **Exposure Cohort** | **Comparison Cohort** | **SMD** |
| Total No. | 35,517 | 33,324 |  | 27,769 | 27,769 |  |
| Age | 59(11.9) | 59.1(11.3) | 0.01 | 59(11.8) | 59(11.4) | 0.0002 |
| ***Sex, %*** | | | | | | |
| Female | 48.8 | 38.8 | 0.20* | 43.2 | 43.3 | 0.003 |
| Male | 46.9 | 56.3 | 0.18* | 52.1 | 51.9 | 0.003 |
| Unknown Gender | 4.3 | 4.9 | 0.03 | 4.7 | 4.8 | 0.0002 |
| ***Ethnicity, %*** | | | | | | |
| Hispanic/Latinx | 8.5 | 10.3 | 0.06 | 9.4 | 9.3 | 0.004 |
| Not Hispanic/Latinx | 67.5 | 64.2 | 0.06 | 65.5 | 65.7 | 0.003 |
| Unknown Ethnicity | 24.0 | 25.5 | 0.03 | 25.1 | 25.0 | 0.001 |
| ***Race, %*** | | | | | | |
| African American/Black | 13.2 | 10.4 | 0.08 | 11.5 | 11.5 | 0.0004 |
| White | 65.3 | 63.9 | 0.02 | 65.3 | 65.4 | 0.001 |
| Asian | 3.5 | 6.2 | 0.12* | 4.2 | 4.2 | 0.0007 |
| ***Adverse socioeconomic determinants of health, %*** | 0.95 | 0.75 | 0.02 | 0.79 | 0.8 | 0.001 |
| ***Problems related to lifestyle, %*** | 2.2 | 2.1 | 0.003 | 2.2 | 2.1 | 0.002 |
| ***Pre-existing medical conditions, %*** | | | | | | |
| Ischemic heart diseases | 14.4 | 16.3 | 0.05 | 14.9 | 14.8 | 0.0008 |
| Other forms of heart disease | 14.6 | 14.7 | 0.0006 | 14.0 | 14.1 | 0.0009 |
| Cerebrovascular diseases | 5.9 | 5.8 | 0.0007 | 5.6 | 5.7 | 0.002 |
| Disorders of thyroid gland | 15.1 | 12.7 | 0.06 | 13.4 | 13.5 | 0.001 |
| Diseases of liver | 3.4 | 3.4 | 0 | 3.3 | 3.3 | 0.001 |
| Diseases of arteries, arterioles and capillaries | 6.8 | 6.3 | 0.01 | 6.3 | 6.4 | 0.003 |
| Diseases of veins, lymphatic vessels and lymph nodes, not elsewhere classified | 3.6 | 3.3 | 0.01 | 3.3 | 3.2 | 0.002 |
| Disorders of gallbladder, biliary tract and pancreas | 2.2 | 2.3 | 0.009 | 2.2 | 2.2 | 0.003 |
| Diseases of appendix | 0.10 | 0.12 | 0.003 | 0.10 | 0.10 | 0.001 |
| Acute kidney failure and chronic kidney disease | 9.0 | 4.7 | 0.17* | 5.7 | 5.6 | 0.006 |
| Renal tubulo-interstitial diseases | 1.9 | 1.7 | 0.01 | 1.6 | 1.7 | 0.005 |
| Glomerular diseases | 1.3 | 0.9 | 0.04 | 0.95 | 0.95 | 0.0003 |
| Urolithiasis | 3.5 | 3.3 | 0.01 | 3.3 | 3.2 | 0.005 |
| Hyperlipidemia | 38.9 | 39.2 | 0.006 | 37.9 | 37.8 | 0.001 |
| Gastroparesis | 0.57 | 0.52 | 0.006 | 0.52 | 0.56 | 0.005 |
| Anorexia nervosa | 0.03 | 0 | 0.02 | 0 | 0 |  |
| Disorders of fluid, electrolyte and acid-base balance | 5.5 | 4.6 | 0.04 | 4.7 | 4.6 | 0.001 |
| Malignant neoplasms of digestive organs | 0.28 | 0.35 | 0.01 | 0.30 | 0.31 | 0.001 |
| Intestinal infectious diseases | 0.80 | 0.67 | 0.01 | 0.65 | 0.69 | 0.004 |
| Noninfective enteritis and colitis | 3.6 | 3.1 | 0.03 | 3.2 | 3.2 | 0.001 |
| Hernia | 2.4 | 2.3 | 0.001 | 2.2 | 2.2 | 0.004 |
| Diseases of peritoneum and retroperitoneum | 0.47 | 0.34 | 0.02 | 0.34 | 0.38 | 0.006 |
| ***Pre-existing procedures, %*** | | | | | | |
| Surgical Procedures on the Colon and Rectum | 0.38 | 0.06 | 0.06 | 0.09 | 0.08 | 0.003 |
| ***Pre-existing anti-diabetic medicine, %*** | | | | | | |
| Insulins | 37.6 | 25.8 | 0.25* | 29.3 | 29.3 | 0.0005 |
| Biguanides | 52.1 | 52.6 | 0.01 | 50.7 | 50.8 | 0.002 |
| Sulfonylureas | 29.9 | 30.1 | 0.003 | 29.1 | 29.3 | 0.002 |
| Alpha glucosidase inhibitors | 0.51 | 0.45 | 0.009 | 0.43 | 0.44 | 0.001 |
| Thiazolidinediones | 9.1 | 7.6 | 0.05 | 8.0 | 8.0 | 0.0002 |
| Dipeptidyl peptidase 4 (DPP-4) inhibitors | 19.3 | 24.5 | 0.12* | 21.0 | 20.9 | 0.003 |
| Other blood glucose lowering drugs, excl. insulins | 2.1 | 1.7 | 0.03 | 1.7 | 1.8 | 0.001 |
| ***Pre-existing other medicine, %*** | | | | | | |
| ACE inhibitors | 33.6 | 32.8 | 0.01 | 32.1 | 32.2 | 0.002 |
| Anti-inflammatory and antirheumatic products | 25.8 | 23.9 | 0.04 | 24.1 | 23.9 | 0.003 |
| Opioids | 26.4 | 24.4 | 0.04 | 24.6 | 24.5 | 0.001 |
| Glucocorticoids | 25.7 | 24.4 | 0.03 | 24.1 | 24.2 | 0.001 |
| Diuretics | 27.6 | 23.4 | 0.09 | 24.4 | 24.4 | 0.0001 |
| Beta blocking agents | 26.9 | 25.6 | 0.02 | 25.2 | 25.3 | 0.0004 |
| Platelet aggregation inhibitors | 27.0 | 25.9 | 0.02 | 25.3 | 25.4 | 0.001 |
| Antihistamines | 20.9 | 18.2 | 0.06 | 18.7 | 18.6 | 0.001 |
| Antidepressants | 22.4 | 16.8 | 0.13* | 18.5 | 18.4 | 0.0008 |
| Serotonin (5HT3) antagonists | 16.6 | 15.9 | 0.02 | 15.7 | 15.8 | 0.001 |
| Drugs for constipation | 17.1 | 15.1 | 0.05 | 15.3 | 15.4 | 0.001 |
| Calcium channel blockers | 17.6 | 15.9 | 0.04 | 15.8 | 16.2 | 0.01 |
| Selective beta-2-adrenoreceptor agonists | 14.8 | 12.3 | 0.07 | 12.9 | 13.0 | 0.001 |
| Anticoagulants | 13.4 | 13.0 | 0.01 | 12.7 | 12.9 | 0.005 |
| Anxiolytics | 13.7 | 11.7 | 0.06 | 12.1 | 12.2 | 0.0004 |
| Anticholinergics | 4.8 | 3.9 | 0.04 | 4.2 | 4.1 | 0.001 |
| Antipsychotics | 4.0 | 2.9 | 0.05 | 3.3 | 3.2 | 0.005 |

Note: SMD - standardized mean difference. *SMD > 0.1, a threshold indicating imbalance between cohorts.

**Figure S1.** Flow diagram of cohort design (GLP-1RAs cohort vs Metformin cohort)


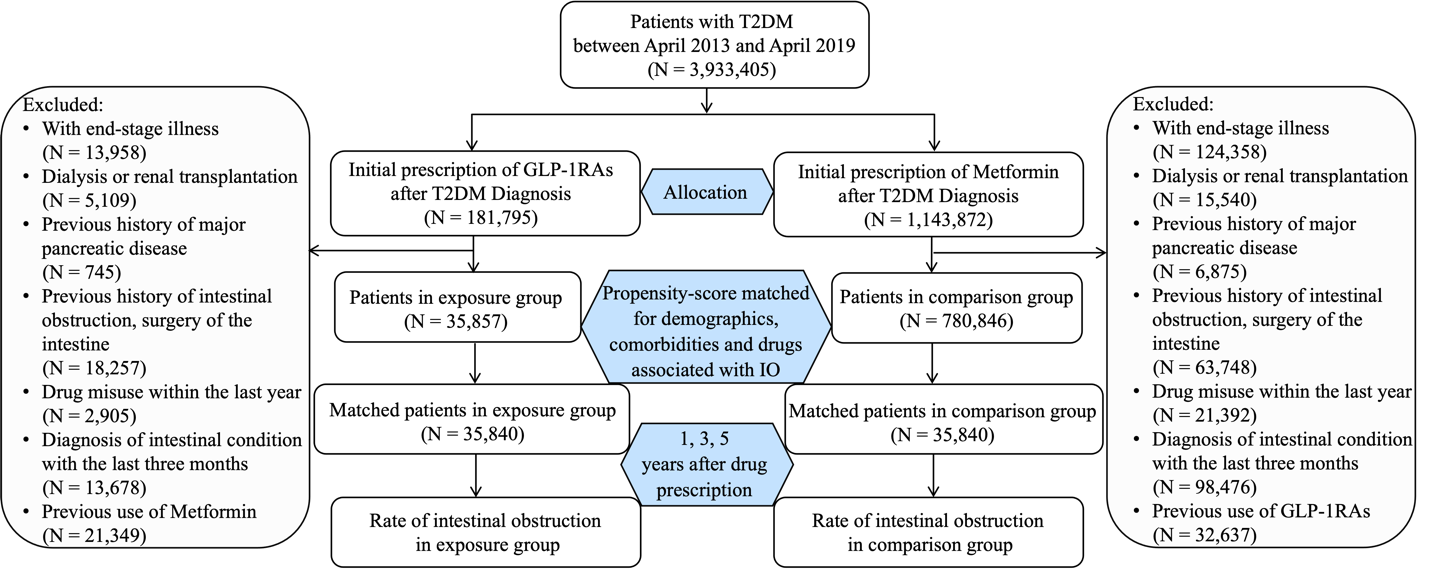


**Figure S2.** Flow diagram of cohort design (GLP-1RAs cohort vs Sulfonylureas cohort)


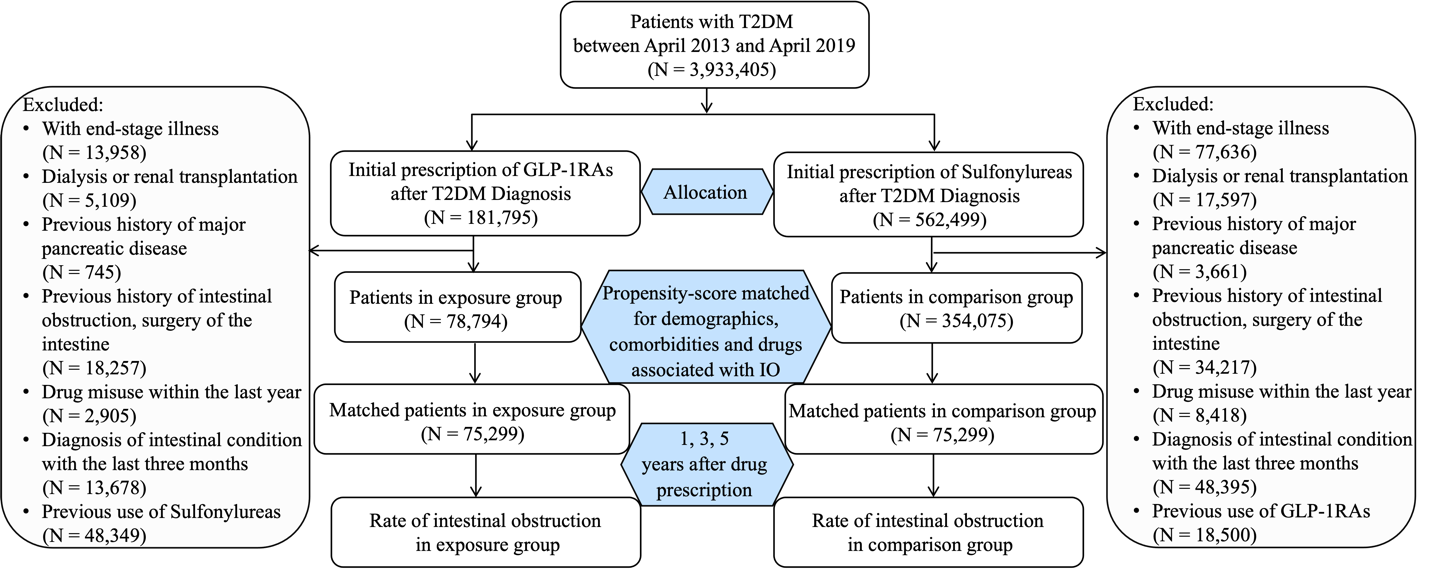


**Figure S3.** Flow diagram of cohort design (GLP-1RAs cohort vs Thiazolidinediones cohort)


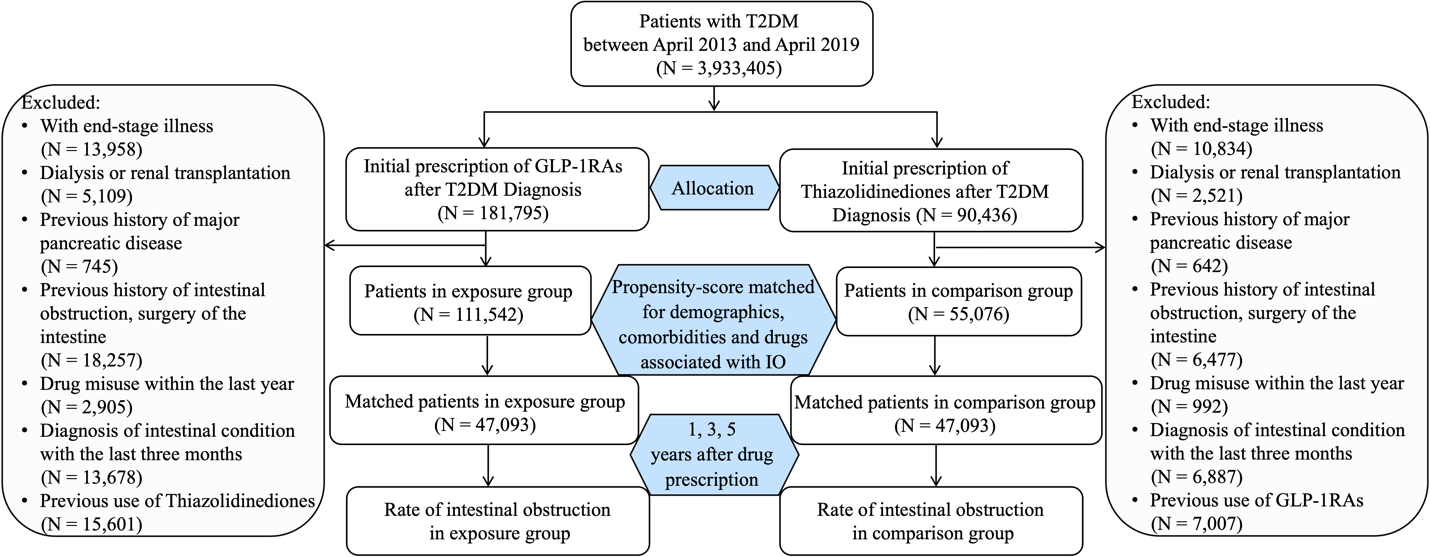


**Figure S4.** Flow diagram of cohort design (GLP-1RAs cohort vs DPP-4 cohort)


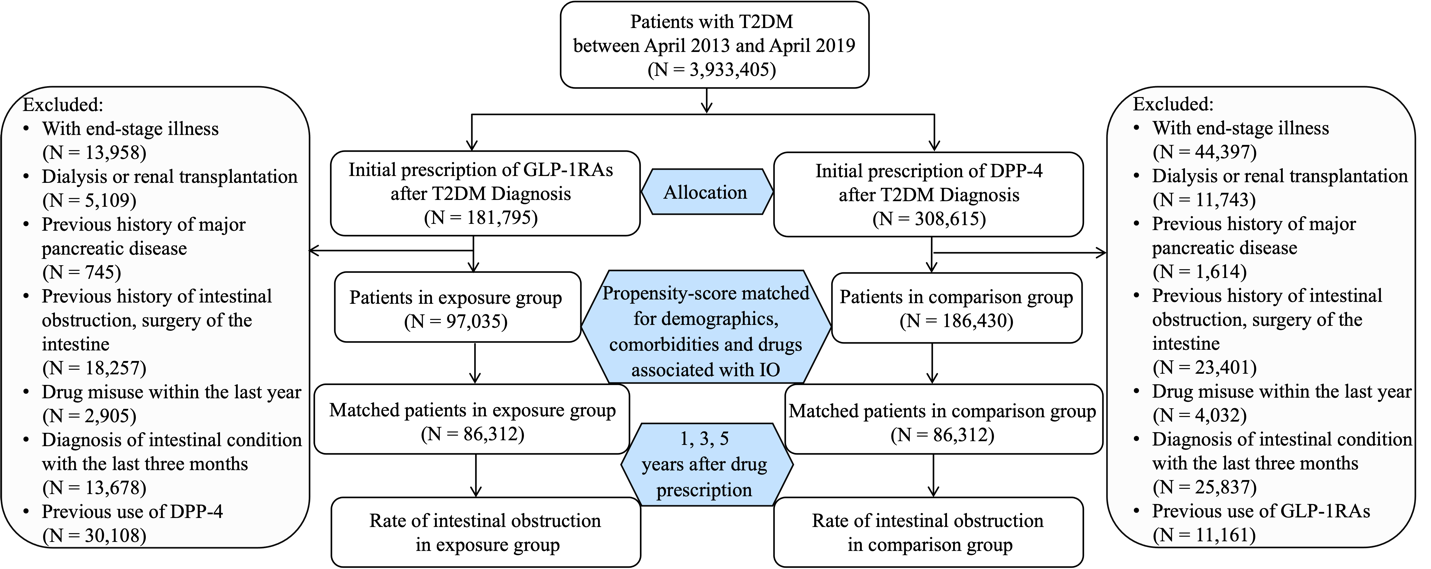


**Figure S5.** Flow diagram of cohort design (GLP-1RAs cohort vs Insulin cohort)


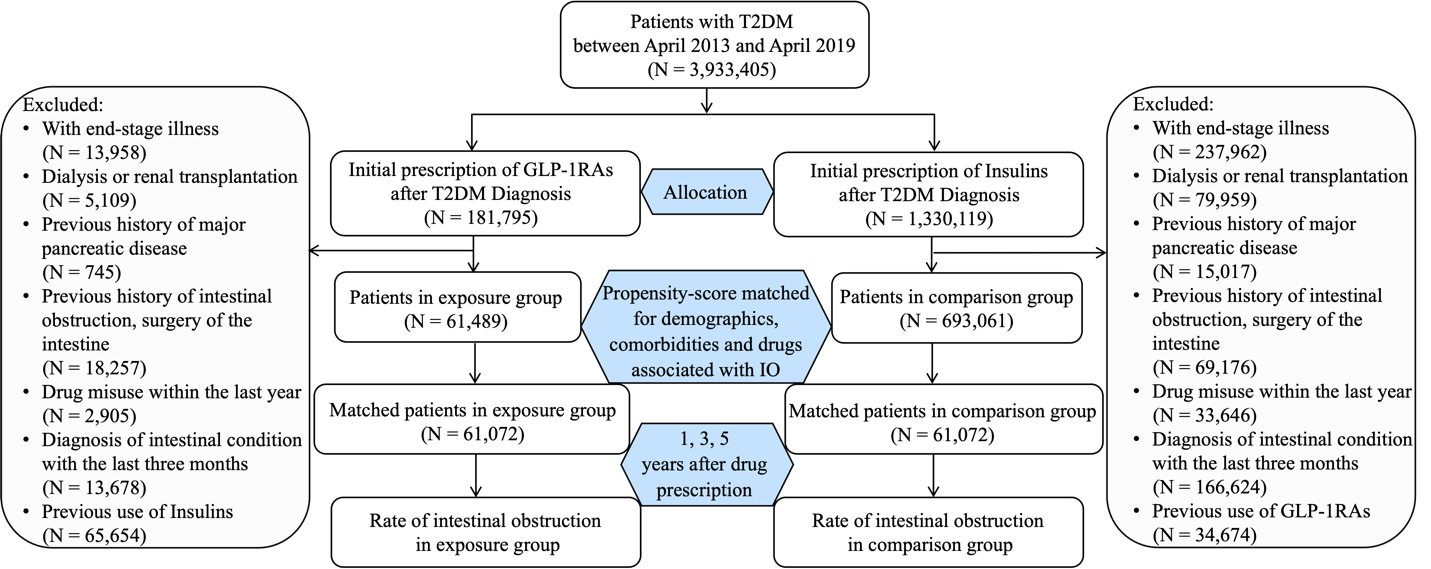


**Figure S6.** Hazard ratios for the diagnosis of subtype intestinal obstruction in T2DM patients prescribed with GLP-1RAs compared with propensity score-matched patients prescribed alternative diabetes medications (Follow up: 5 years)


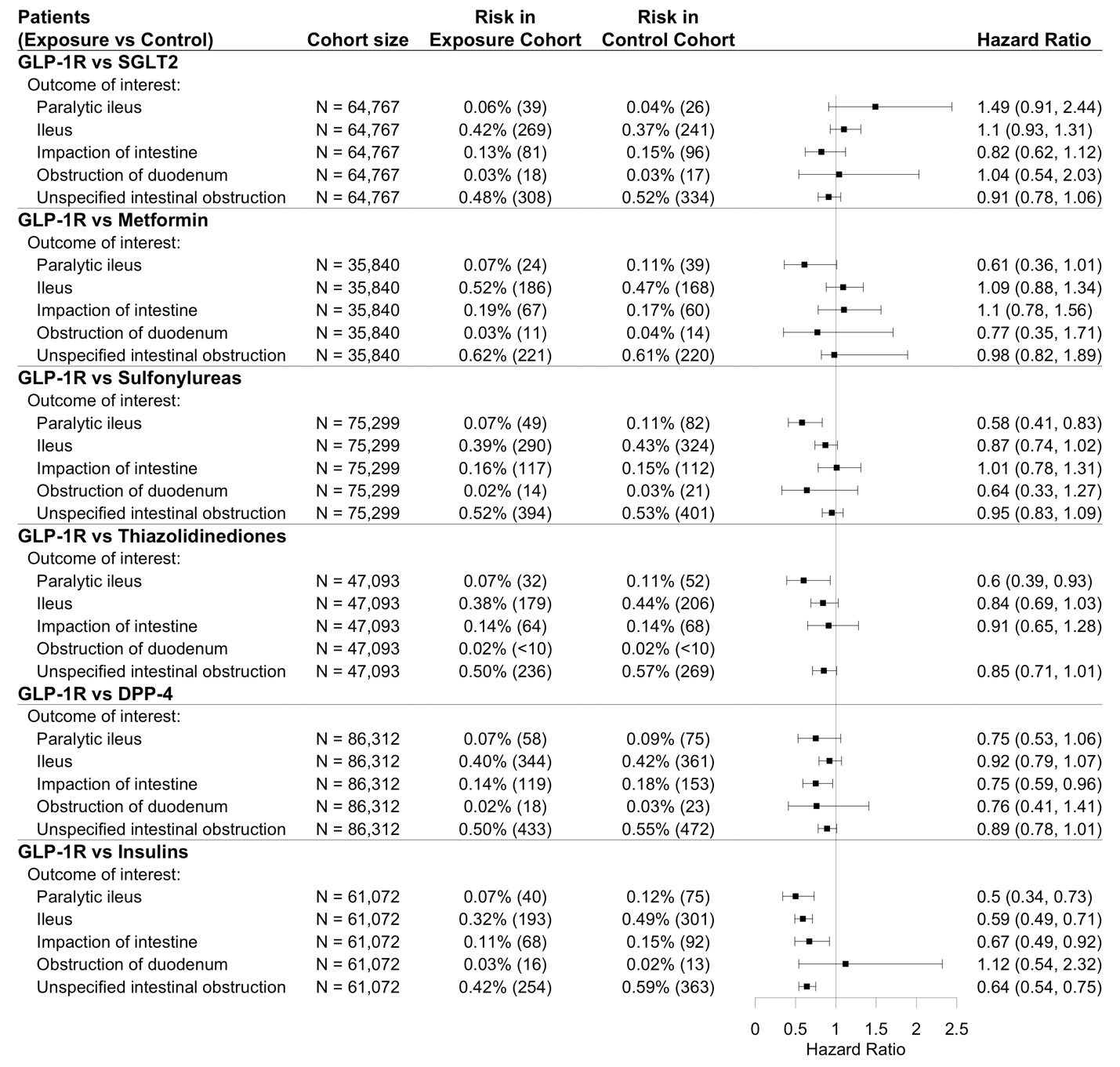


**Figure S7**. Subgroup analysis focusing on patients aged 65 years and older showed results consistent with the main analysis (Follow up: 5 years).


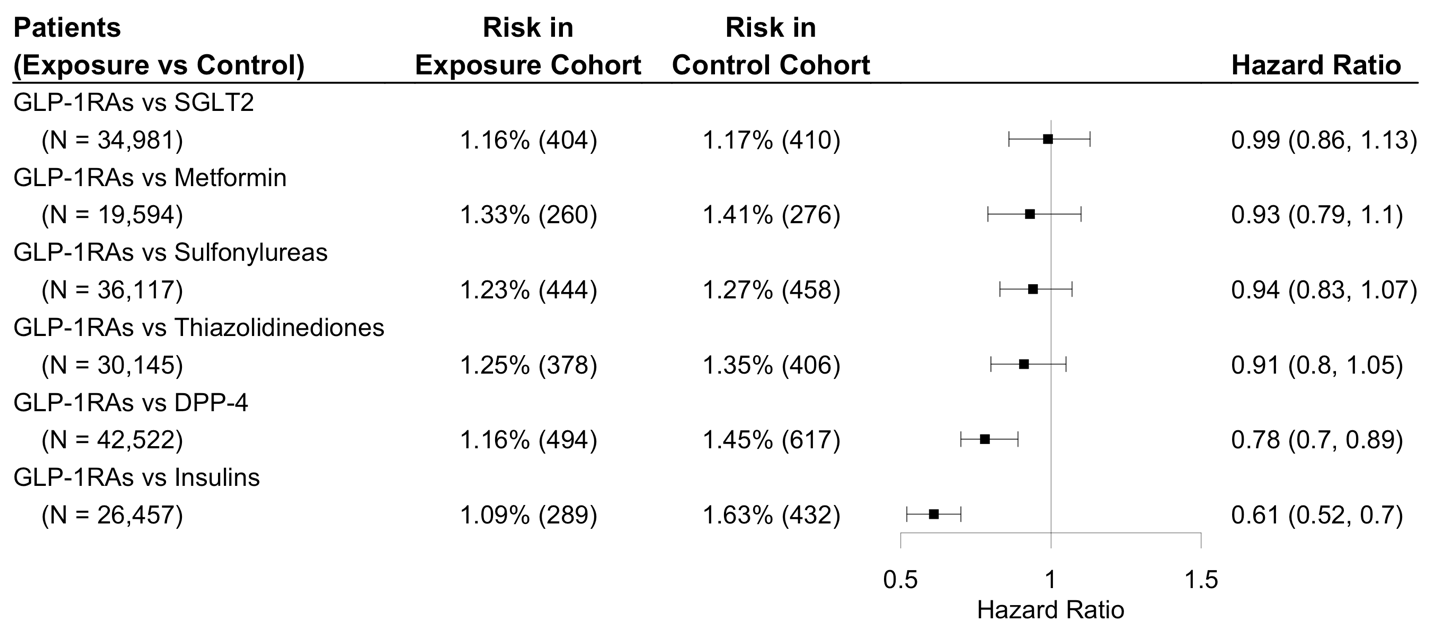


**Figure S8.** Sensitivity analysis excluding patients with a history of alpha-glucosidase inhibitor use from both the Exposure and Comparison Cohorts. The analysis demonstrates consistent results with the primary findings (Follow up: 5 years).


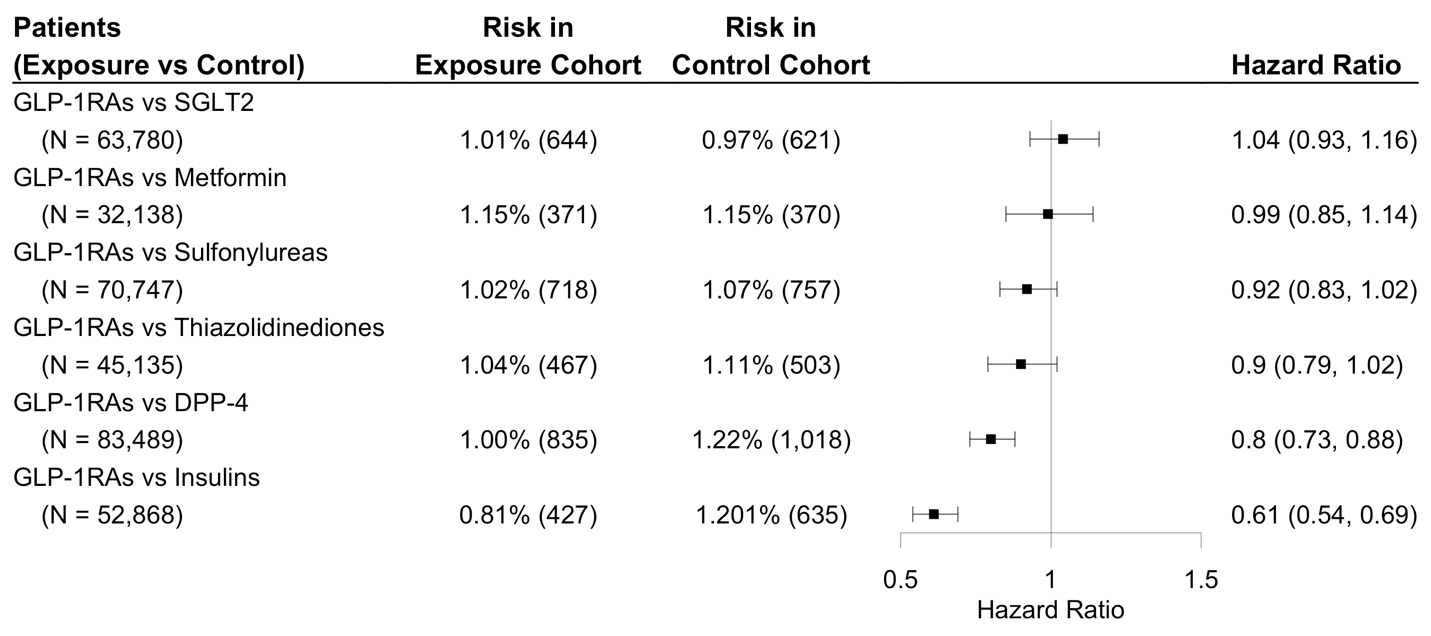


**Figure S9.** Hazard ratios for the diagnosis of subtype intestinal obstruction in T2DM patients with obesity prescribed with GLP-1RAs compared with propensity score-matched patients prescribed alternative diabetes medications (Follow up: 5 years)


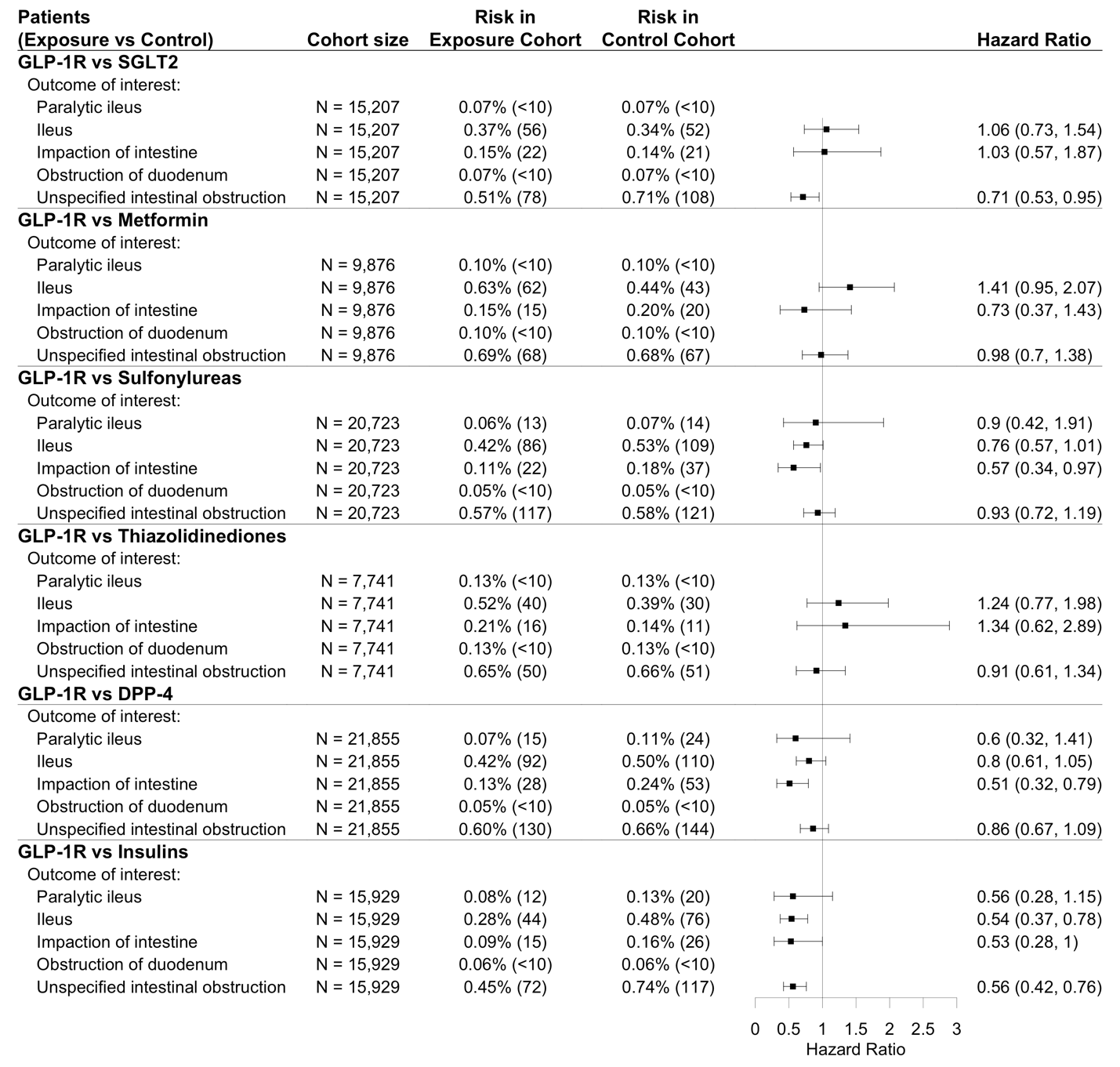


**Figure S10.** Hazard ratios for the diagnosis of subtype intestinal obstruction in T2DM patients without obesity prescribed with GLP-1RAs compared with propensity score-matched patients prescribed alternative diabetes medications (Follow up: 5 years)


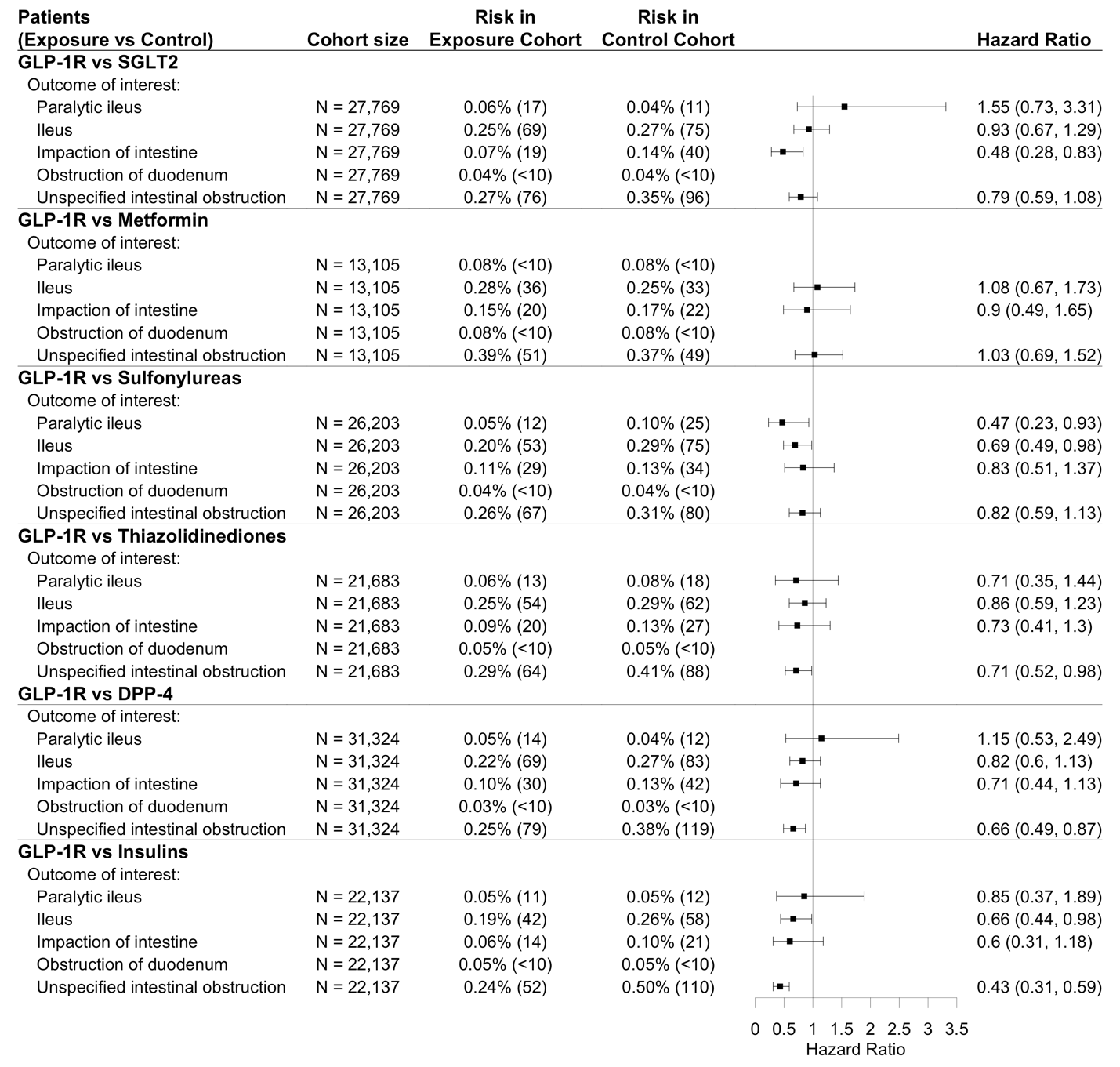

Supplement: Supplementary file 2 — Supplementary Material 2 [file 592_2025_2525_MOESM2_ESM.docx]
